# Testing the effect of an integrated-intervention to promote access to sexual and reproductive healthcare and rights among women with disabilities in Ghana: a quasi-experimental study protocol

**DOI:** 10.1186/s12978-021-01253-1

**Published:** 2021-10-15

**Authors:** John Kuumuori Ganle, Charlotte Ofori, Samuel Dery

**Affiliations:** 1 Department of Population, Family and Reproductive Health, School of Public Health, University of Ghana, Legon, Accra, Ghana; 2 Regional Institute for Population Studies, University of Ghana, Legon, Accra, Ghana; 3 Department of Biostatistics, School of Public Health, University of Ghana, Legon, Accra, Ghana

**Keywords:** Disability, Women with disability, Reproductive health, sexual health, Rights, Access, Ghana

## Abstract

**Background:**

There is evidence that women with disabilities (WWDs) experience the most difficulty accessing and using sexual and reproductive health and rights (SRHRs) services and information worldwide. However, there are currently no workable interventions to reach WWDs with essential SRHR services. This study aims to test the effect of an integrated health facility and individual-level intervention on access to SRHRs information and services among sexually active WWDs aged 15–49 years in Ghana.

**Methods:**

A quasi-experimental study design with four arms will be implemented in four districts in the Northern region of Ghana to test the effect of three inter-related interventions. The inventions are (1) capacity building in disability-centred SRHRs information and service delivery for healthcare providers, (2) support for WWDs to access disability-unfriendly healthcare infrastructure, and (3) one-on-one regular SRHRs education, information provision, and referral. The first two interventions are at the health-facility level while the third one is at the individual/family level. The first arm of the experiment will expose eligible WWDs to all three interventions. In the second arm, WWDs will be exposed to only the two-health facility-level interventions. The third arm will expose WWDs to only the individual level intervention. The forth arm will constitute the control group. A total of 680 (170 in each arm) sexually active women with physical disability and visual impairments will take part in the study over a period of 12 months. To assess the effect of the interventions on key study outcomes (i.e. awareness about, and use of modern contraceptive, ANC attendance, and skilled delivery among parous women), pre- and post-intervention surveys will be conducted. Difference-in-Difference analysis will be used to examine the effect of each intervention in comparison to the control group, while controlling for confounders. Cost-effectiveness analyses will also be conducted on the three-intervention arms vis a vis changes in key outcome measures to identify which of the three interventions is likely to yield greater impact with lower costs.

**Discussion:**

Lack of access to SRHRs information and services for WWDs is not only a violation of their right to appropriate and quality SRH care but could also undermine efforts to achieve equitable healthcare access as envisaged under SDG 3. This research is expected to generate evidence to inform local health programmes to increase access to SRHRs among WWDs by strengthening local health system capacity to provide disability-sensitive SRHRs services.

*Trial registration *Name of the registry: Pan African Clinical Trials Registry (PACTR). Trial ID: 14591. Date of registration: 02/01/2020. URL of trial registry record: https://pactr.samrc.ac.za/Researcher/TrialRegister.aspx?TrialID=14591

## Plain English summary

Women with disabilities (WWDs) experience a lot of difficulty accessing sexual and reproductive health and rights (SRHRs) services and information worldwide. However, there are currently no workable specific interventions to reach WWDs with essential SRHRs services. We propose to implement three interventions to enable WWDs in Ghana who are aged 15–49 years gain better access to SRHRs services and information. Our aim is show whether these interventions have beneficial effects by helping many more WWDs access and use SRHRs services and information like modern contraceptives. To achieve this objective, we will (1) train healthcare providers in ways to better provide SRHRs information and service to WWDs; (2) support WWDs to access disability-unfriendly healthcare infrastructure by creating access ramps at health facilities; (3) train and deploy community health volunteers to undertake one-on-one regular home visits to educate WWDs on SRHRs, provide information on SRHRs and refer WWDs to healthcare providers. A total of 680 sexually active women with physical and visual impairments will take part in the study over a period of 12 months. We will divide the women into four groups (170 in each group). The first group will be exposed to all the three interventions. The second group will be exposed to only interventions 1 and 2. The third group will be exposed to only intervention 3. The fourth group will not be exposed to any of the three interventions. Our study is expected to strengthen the local health system capacity to provide disability-sensitive SRHRs services.

## Background

Within the last three decades, much progress has been made to improve sexual and reproductive and rights (SRHRs) worldwide. As a result, global maternal mortality ratio has decreased from 380 to 210 per 100,000 live births between 2000 and 2013 [1]. This progress notwithstanding, many women still lack access to life-saving sexual and reproductive health (SRH) services in many low-income countries, including Ghana. One group that has particularly been disadvantaged but has nevertheless received little attention in low-income settings is people with disabilities (PWDs). According to the *World Report on Disability 2011*, disability is the umbrella term for impairments, activity limitations and participation restrictions, referring to the negative aspects of the interaction between an individual (with a health condition) and that individual’s contextual factors (environmental and personal factors) [2]. Globally, PWDs constitute 15% of the world’s population, of which over 80% lives in low-income countries [2]. Persons with disabilities remain one of the most marginalized and socially excluded groups, and this disadvantage transcends several spheres: PWDs have generally poorer health, lower education achievements, fewer economic opportunities and higher rates of poverty than people without disabilities [2–4]. In particular, women with disabilities are more likely to be poorer and have lower social and economic status than their counterparts who have no disability [3, 4].

In the context of sexual, reproductive, and maternal health more specifically, a number of recent studies note that women with disabilities have largely been ignored in research and programming [2, 5–17]. Part of the reason for this neglect is that women with disabilities are often thought not to be sexually active, and less likely to marry or to want to have children than non-disabled women [5–17]. Others attribute this neglect to a complex web of discrimination made up of negative social attitudes and cultural assumptions as well as environmental barriers including policies, laws, structures and services, which result in marginalization and social exclusion [6–18]. Although attitudes may be changing in some contexts [17], stigma and prejudice against women with disability often prevent them from accessing sexual, reproductive, and maternal healthcare information and services [18]. Meanwhile, recent data shows that rates of sexual activity, need for family planning (FP), and childbirth services among women with disabilities are comparable to those of non-disabled women [19–21]. This challenge is compounded by the fact that research on disability and reproductive health is even far more limited in many low-income contexts, including Africa [5, 22–24]. A recent study puts this issue quite bluntly: ‘There is a complete lack of published literature in peer reviewed journals on the reproductive health status of women with disabilities’ [25].

Ghana, like many countries in sub-Saharan Africa (SSA), has made progress over the last several decades to improve the health of its citizens. Despite this progress, Ghana’s health sector still faces critical healthcare service delivery challenges in the areas of sexual reproductive and maternal health, including low use of modern contraceptives. Data from Ghana’s 2014 Demographic and Health Survey suggest that while knowledge about modern contraceptive methods is universal (> 99%), only 27% of married women are using any contraceptive method [26]. Unmet need for family planning among married women is still 30%. There are however local and regional variations, with the Northern Region (where this study will be implemented) having the highest unmet need for birth spacing (21.7%). Similar disparities exist for skilled attendance at birth: at the national level, 74% of births are attended by skilled personnel compared to 36.4% of births in the Northern region [26]. At the same time, traditional birth attendants delivered some 41% of births in the Northern Region [26].

One group that has particularly been disadvantaged but has nevertheless received little attention in Ghana is persons with disabilities [22, 24]. Approximately 3% of Ghana’s population has some disability [27]. This prevalence however varies from one geographic location to another and across different socio-economic groups. For instance, recent estimates suggest that among women of reproductive age (15–49 years) in Ghana, 10.2% have some kind of disability [31]. The three most prevalent types of disability in Ghana are those related to physical disabilities, visual impairment, and hearing impairment [27]. Although Ghana is a signatory to the United Nations Convention on the Rights of Persons with Disabilities [28], and also enacted the Persons with Disability Act (Act 715) in 2006 [29], widespread enforcement of these laws is yet to be reported. Indeed, previous research by Ganle and colleagues have highlighted critical challenges women with disability still face in accessing modern contraceptive and skilled maternal healthcare services in Ghana [22]. For instance, the authors showed that many women with disabilities who are sexually active, and do want to receive contraception and skilled maternal healthcare information and services often encounter grave barriers, including healthcare providers’ insensitivity and lack of knowledge about the sexual health, family planning and maternity care needs of women with disabilities; unfriendly or inaccessible healthcare infrastructure; health information that lacks specificity in addressing the special sexual and reproductive health needs of women with disabilities; and lack of social support at community and health facility levels to assist women with disability access unfriendly physical health infrastructure to receive appropriate sexual and reproductive health information and services [22]. Consequently, many women with disabilities often turn to other sources of care, including use of untrained traditional birth attendants during childbirth [22].

Recent global emphasis on the rights of PWDs in the United Nations Convention on the Rights of Persons with Disabilities as well as current emphasis on shared economic growth and prosperity under the Sustainable Development Goals have re-emphasized the necessity of addressing disabled women/girls’ sexual and reproductive health rights. What is currently lacking in many African contexts, however, is how to innovatively address the sexual and reproductive health and rights needs and challenges of women with disabilities. Although there are some interventions in high-income settings [25, 30], this is not the case in many settings in Africa, including Ghana. Currently, targeted and proven interventions to reach women with disabilities with essential sexual reproductive health services are lacking [30]. To address this challenge, the current study proposes to implement an integrated set of interventions to address some key barriers women with disability face accessing sexual and reproductive health and rights information and services in Ghana. Specifically, the study seeks to (i) determine awareness and use of sexual and reproductive health services among women with disabilities before intervention; (ii) estimate and compare the effect of exposing women with disability to different components of an integrated intervention on awareness about, and use of family planning/modern contraceptive services, antenatal care attendance and skilled birth services; (iii) identify other significant determinants of SRH care services utilization among WWDs; and (iv) assess the cost-effectiveness of implementing either the integrated package of three interventions, or only the health facility or individual level interventions.

## Methods

### Context

This research will be implemented in the Northern region of Ghana. As indicated earlier, the region has one of the highest unmet need for sexual and reproductive health services and the lowest supervised delivery rates in the country. For instance, while 25% of married women aged 15–49 years in Ghana used modern contraceptives in 2017, only 16.8% did so in the region [31]. The project would be implemented in four districts with the highest disability rates in the region: Central Gonja (3.6%), West Gonja (3.8%), Savlugu (4.6%), and Bunkprugu-Nankpanduri (5.4%) districts [32]. While the four districts chosen for the experiment are geographically distant enough to limit cross-contamination of participants, the districts are nevertheless socially, culturally and economically similar. This similarity will ensure comparability of results.

### Design

A quasi-experimental quantitative study design, involving pre- and post-intervention surveys, will be used to test the effect of three inter-related interventions. Although evidence from designs such as cluster randomized clinical trial is typically at a much higher level than a quasi-experimental study, we chose a quasi-experimental design because the four study districts were purposively selected. Therefore, it was not possible to randomize the districts as clusters.

The inventions are *(1) capacity building in disability-centred sexual and reproductive health and rights information and services delivery for healthcare providers; (2) support for women with disabilities to access disability-unfriendly healthcare infrastructure and (3) one-on-one regular sexual and reproductive health and rights education, information provision, and referral*. While detailed description of each intervention is provided later, it is important to specify that the first two interventions are at the health-facility level while the third one is at the individual/family level.

In this quasi-experimental design, there are four arms; each to be based in one district determined using a simple random sampling procedure. In the first arm, eligible women with disabilities in the Savelugu Municipality will be exposed to all the three interventions. In the second arm, eligible women with disabilities in Bunkprugu-Nankpanduri district will be exposed to only the two-health facility-level interventions. The third arm (in Central Gonja District) will be exposed to only the individual level intervention. The forth arm (based in West Gonja district) will constitute the control group (i.e. women with disabilities will not be exposed to any intervention). To assess the effect of each of the three arms of interventions, we will first conduct a cross-sectional pre-intervention survey among all women with disabilities in each intervention arm. This survey will aim to collect baseline data on several outcomes including awareness about modern contraceptives, use of modern contraceptives, including antenatal care attendance and skilled delivery among those who have given birth before. The same survey will be repeated at the end of the intervention (i.e. 12 months) on the same women in all arms of the experiment.

### Description of inventions

The three interventions would be implemented over a period of 12 months. The interventions have been developed based on the barriers women with disability face in accessing sexual and reproductive healthcare that Ganle and colleagues have identified in Ghana and sub-Saharan Africa through primary research [22] and a systematic review [5].

#### Capacity building in disability-centred sexual and reproductive health and rights information and service delivery for healthcare providers

The first health system level intervention is *capacity building in disability-centred sexual and reproductive health and rights information and service delivery for healthcare providers.* A major barrier to uptake of sexual and reproductive health services by women with disabilities in Ghana and elsewhere is healthcare providers’ lack of knowledge about the sexual and reproductive health needs of women with disability [5, 22]. This situation results partly from lack of formal training in the provision of sexual and reproductive services to women with disabilities [22]. For example, Ghana’s Disability Act mandates the study of disability issues in the curricula of training institutions for health professionals to develop appropriate human resources to provide general and specialized care services to PWDs [29]. However, this has yet to happen in many health training institutions [22]. To address this challenge, a one-time 2-day capacity building training in disability-centred sexual and reproductive health information and service delivery for frontline health workers (e.g. midwives and community health nurses) under the Ghana Health Service and Christian Health Association of Ghana will be organized. The aim is to equip sexual and reproductive healthcare providers with basic knowledge and skills to be able to provide need-based sexual and reproductive health information and services to women with disabilities.

The training will have two components: enhancing service providers’ understanding of disability issues, and appropriate sexual and reproductive health services including family planning and contraceptive methods for disabled women/girls. In relation to the first component, the training will focus on imparting into healthcare providers (i) basic knowledge of disability and how to recognize it in its various forms; (ii) health information tailored to address the special sexual health, family planning and maternity care needs of women with disabilities; (iii) use of respectful and appropriate disability terminologies and relevant communication skills when dealing with women with disabilities within health facilities; and (iv) technical skills and abilities to enable frontline healthcare providers evaluate the needs of every woman with disability before recommending and/or providing appropriate sexual and reproductive health services.

As regards the second component, the training will focus on educating service providers on the different sexual and reproductive health needs and challenges of women/girls with disabilities. In particular, the training will highlight how specific family planning/contraceptive services could be safely and appropriately provided to women/girls with different disabilities. This component of the training will also expose healthcare providers to global best practices in disability-centred care and the ways in which these could be adopted or modified and applied in the Ghanaian context.

We expect to train 30 service providers from each of the two districts (i.e. Savelugu Municipality and Bunkpurugu-Nankpanduri districts) in which this health system level intervention will be implemented. Mechanisms for monitoring training effectiveness will include before and after training surveys to compare awareness and knowledge on disability issues, changes in attitudinal biases, and regular monitoring at service delivery points to evaluate how knowledge and skills acquired from the training are being translated to enhance service delivery.

#### Supporting disabled women/girls to gain access to less-disability friendly healthcare infrastructure

The second facility-level intervention we propose is to implement a relatively low-cost intervention at existing health facilities to support women with physical and visual disabilities gain access to inaccessible healthcare infrastructure. We recognize that capacity building in disability-centred health information and care delivery for healthcare providers may not bring about the needed change if women with disabilities are unable to gain access to these trained healthcare providers. We therefore propose to implement a relatively low-cost but potentially effective and sustainable intervention at existing health facilities to help support women with disabilities gain access to less-disability friendly healthcare infrastructure.

This intervention is directly informed by findings from previous research in Ghana, which suggest that most healthcare facilities currently lack appropriate ramps, wheelchairs, and personnel to assist women with disabilities, especially pregnant women climb stairs, examination tables and delivery beds [5, 22]. These problems often combine to discourage some women with disabilities from seeking skilled care [5, 22]. Given that sexual and reproductive health for women with disabilities are often contentious in many communities in Ghana [22], relevant support from family members may be lacking for many disabled women/girls. Therefore, the idea is to train and designate at least one frontline, low-skilled auxiliary health worker at every health facility that provides sexual and reproductive health services in the two districts where health facility-level interventions will occur. Such a person will be responsible for supporting disabled women/girls access buildings and consulting rooms to receive sexual and reproductive health information and services. To minimize cost, promote task-shifting and ensure sustainability, we propose to train existing Medical Orderlies or Ward/Nurse Assistants, and task them with the special additional responsibility of assisting women with disabilities gain access to health facilities. In furtherance of this, we propose to supply low-cost flat wooden or mental boards that are long, wide and strong enough to support women with disabilities on wheelchairs gain access. The idea is to equip facilities, especially those that currently have less-disability friendly stairs, with these boards to help connect the ground unto raised platforms. Trained Orderlies would be required to mount these boards over which women with disabilities on wheelchairs may ride to access facilities. We however expect to use concrete to create access ramps in health facilities where it will be impossible to use these removable mental or wooden mount boards. In each of the two districts where this intervention will be implemented, we expect to cover not more than 17 health facilities.

#### One-on-one regular sexual and reproductive health and rights education, information provision, and referral

Support for women with disability at the health facility level is essential but not sufficient to guarantee full access to needed sexual and reproductive health information and services. As in many low-income settings, disability still carries social stigma in Ghana [22, 24]. This often results in limited social support for women with disabilities, including support to receive healthcare [5, 24]. We propose to implement a one-on-one regular sexual and reproductive health education and information provision, and referral system to help create a supportive system to help women with disabilities access and use sexual and reproductive health services. While a community-wide approach is appealing, evidence from previous research in Ghana show that most decision-making around sexual and reproductive health among women with disabilities revolves around individual disabled women and their families [22, 24]. The idea, therefore, is to match all women with disabilities enrolled either into the first arm of the experiment or the third arm to trained community health volunteers. The aim is to create a local *Buddy System* to support women with disabilities access sexual and reproductive health information and services. This approach is inspired by the success of the birth companion model currently being implemented in a number of low-income settings [33–35].

Community health volunteers are local community members who have already been recruited and trained by the Ghana Health Service to perform auxiliary duties: basic health education (e.g. malaria and cholera), reporting the birth/death of a child or disease outbreaks in their community [36]. As community health volunteers live in communities with women with disabilities and also, have connections to the formal health system, we propose to train and use them. The community health volunteers will be expected to assist women with disability, especially those with visual impairments and mobility challenges, arrange appropriate transportation to travel to healthcare facilities. Also, because most healthcare facilities in Ghana do not currently operate a booking system, and because women with disabilities may require specialized care, community health volunteers would be expected to liaise with individual women with disability, especially pregnant women who may want to deliver in health facilities, to identify dates/times that they (women with disabilities) may want to visit the health facility. Once this information is obtained, the community health volunteers would communicate this to the relevant health facility. This prior notification is important both in terms of avoiding unnecessary delays and also preparing adequately to receive the women with disabilities, including arranging for a trained caregiver and orderlies to be available. Finally, and as a way to promote task shifting, the trained community health volunteers will deliver specific family planning/contraceptive services to women with disability such as condoms, sexual reproductive health information and education such as how to use a condom as well as information about where women with disabilities may obtain more specific services.

All women with disability enrolled into arms 1 and 3 of the study will be visited once every month (12 visits in total) by their respective community health volunteer buddy. Each visit will last for a minimum of 30 min and a maximum of 45 min. To ensure uniformity, each visit will be structured around a specific theme/issue. We will develop and equip the community health volunteers with a simple manual with relevant short educational messages to be delivered during each visit, including messages on type of family planning/contraceptive services women with disabilities could use, as well as where to obtain such services. The content in relation to family planning and other sexual and reproductive health services in the manual will be based on standard Ghana health Service and Ministry of Health approved guidelines and training materials. During these visits, community health volunteers will provide basic sexual and reproductive health information and education to women with disability and as well work with them or their family (especially spouses/partners/guardians) to identify unique health and sexual and reproductive health needs and challenges. These visits and education activities will be monitored using field visit record cards, field supervisors and reports from individual women with disabilities and their families.

In total, 80 community health volunteers (40 in each of the two districts) will be involved in the 1st and 3rd intervention arms. To facilitate communication between community health volunteers, trained sexual and reproductive health service providers and women with disabilities, we will purchase and equip each community health volunteer with a cell phone with monthly call credit. Each community health volunteer will also receive a motorbike to facilitate travel to visit women with disabilities. Finally, community health volunteers will receive a monthly payment (not exceeding US$25) to motivate them to continue to support women with disabilities.

### Study participants

The participants in the pre- and post-intervention surveys will be sexually active women with disabilities in their reproductive age (i.e. 15–49 years). Specifically, women with moderate to severe physical disability (including albinism and leprosy) and visual impairments will be included. We focused on physical and visual impairments partly because they are the dominant disabilities in Ghana [27]. We exclude hearing/speech impairments and mental disabilities partly because deaf and mentally-ill women/girls have needs that are unique and often require further targeting and specialized services (e.g. sign language) that maybe impossible to implement within the time allowed for this project.

### Sample size estimation

To estimate a minimum sample size that will allow for small effect sizes to be detected, we used the sample size estimation formula below:

$$n=Deff\times \frac{{\left\{Z_{1-\beta }\sqrt{ [p_{1}(1-p_{1} )+p_{2}(1-p_{2}) ]}+{Z}_{1-\alpha/2}\sqrt{\left[2\overline{p }(1-\overline{p })\right]}\right\}}^{2}}{(1-NR)\times {(p_{1}-p_{2})}^{2}} {Z}_{1-\alpha / 2}$$

where ${\overline{p}}={\frac{{p_1}+{p_2}}{2}}$; ${p}_{1}=13.1\%,$ which is the anticipated population proportion of modern contraceptive use at baseline based on a recent community-based cross-sectional study among women with disability in Ethiopia that estimated modern contraceptive use prevalence (a key outcome in the current study) [37]; ${p}_{2}=25.1\mathrm{\%},$ which is the expected prevalence of modern contraceptive use after the implementation of the intervention; ${Z}_{1-\alpha/2}$ in the formula is the standard normal variate at 5% level of significance; $NR=10\%$ is the anticipated non-response/loss to follow-up rate; Deff = 1.1 to adjust for design effect.

Based on these assumptions, the estimated sample size for each arm is 170. As the design contains four arms, the sample ratio is 1:1:1:1. This means that 170 women with disabilities will be recruited into each arm of the study, totalling 680.

### Sampling and recruitment

A multistage simple random sampling technique will be used to select 170 eligible women with disabilities from each of the study districts. This will involve several steps as described below.

#### Identification and enumeration

To ensure that women with disabilities from different backgrounds (e.g. rural vs. urban, and literate vs. non-literate) are represented in our study, we will conduct a comprehensive community level identification and enumeration of all sexually active reproductive aged women who identify themselves or are identified by family members as having some disability. This community level enumeration is important because although the Ghana Federation of Disability Organisations, local district assemblies, and other disability groups often have some database of registered members, our engagement with leaders of various disability groups in the study districts showed that this data is not sufficient as many persons with disabilities are often not registered with either the district assembly or the Ghana Federation of Disability Organisations. Therefore, a community level enumeration allows for identification of all potentially eligible participants.

To effectively undertake the community level enumeration, community health volunteers will be engaged in each of the study districts to do a house-to-house listing of all women with disabilities aged 15–49 years who have physical or visual disabilities. We propose to use community health volunteers because disability in Ghana still carries considerable stigma such that using a ‘stranger’ to do this household enumeration may be less successful [22, 24]. However, as community health volunteers are community members who may be well known and trusted because of the roles they already play in serving as a link between the formal healthcare system and local communities, women with disabilities and their families are likely to better cooperate.

We will design a simple *Enumeration Form* and train community health volunteers to use the form to enlist all women with physical and visual disability in their respective communities. The form will ask for basic information such as name, community, age, contact number, house number/name, disability type, and whether the disabled woman/girl is sexually active (i.e. have had penetrative sex before). Each of the women with disabilities would also be asked if she would be interested in taking part in the larger study, which will last for a period of 12 months.

In addition to using the enumeration form, we will also train the community health volunteers to administer an adapted screening tool from the Washington Group on Disability Statistics [6]. This screening tool has been successfully used in other low-income contexts to screen and identify women with disabilities [6]. The tool has 35 questions to detect disability type and social function and communication disabilities based on the International Classification of Functioning, Disability and Health [6]. The screening tool captures severity of disability by asking respondents to rank their status on a four-point Likert scale [6]. This tool will allow us to not only identify women/girls with disability but to determine the severity of their disability.

#### Screening

Following the identification and enumeration of all women with physical and visual disabilities in the study districts, we will screen to identify potentially eligible participants. The key considerations will be age (i.e. 15–49 years), disability type (i.e. physical, including albinism and leprosy, and visual disability), and whether the disabled woman/girl has had penetrative sex before.

#### Sampling

Following from the screening, we will create a register of all eligible participants in each district, subdivided along the lines of the two main forms of disabilities described earlier. Depending on the number of eligible participants in each category of disabilities, the total sample size of 170 per district will be proportionately divided among the different categories of disabilities. This means that sampling will be done proportionate to size, so that categories of disabilities with higher numbers of eligible participants are sampled more into the survey. Following from this, each of the eligible participant in each district and disability type will be given a unique number identifier (e.g. 001…00n). The numbered lists will then be imported, one list after the other, into a R statistical software, where the required number of respondents from each district (i.e. 170) and category of disability will be randomly selected.

#### Recruitment

In terms of the procedure for contacting and recruiting eligible women with disabilities into the survey, we will use the contact details collected during the enumeration phase alongside help from community health volunteers to contact all selected women with disabilities in their communities to further discuss the purpose of study and invite them to participate. Following this initial contact, each participant will be given 1 week to make a final decision on whether to participate in the study. They will each be re-contacted via telephone or other appropriate means after the 1-week period. All participants who will decline participation will be dropped and the sampling procedure repeated to get replacement from any remaining eligible potential participants. Where the list of eligible participants for any of the study districts is fully exhausted, there shall be no replacement.

### Data collection

A face-to-face interview method will be used to collect data from individual participants. A total of eight graduate research assistants will be recruited and trained to collect the data. Where feasible, all the research assistants will be persons with disabilities, and will be speakers of at least one of the local languages (i.e. *Dagbaani, Gonja* or *Moar*) in addition to English. They will also have previous experience of collecting survey data.

A structured questionnaire will be developed, pre-tested and used for data collection. The questionnaire will be developed in English, but the questions may be translated and asked in one of three local languages (i.e. *Dagbaani, Gonja* or *Moar*). The questionnaire will collect information to address the research objectives related to assessment of knowledge about modern family planning/contraceptives, and family planning/contraceptive use. Also, sociodemographic information of each respondent will be collected.

Several validated questions from the Ghana Demographic and Health Survey questionnaire [26] and the Ghana Maternal Health Survey questionnaire [31] will be used. These questions are related to the key outcome variable for the proposed study which is contraceptive use/uptake. Further, the questions will also assess awareness and knowledge of contraceptive use, contraceptive use negotiation, safe sex negotiation, use of multiple methods, use of antenatal care, and skilled delivery care services for respondents who have been pregnant or are currently pregnant as well as those who have given birth before. With regard to the primary outcome variable, contraceptive use/uptake, participants will be asked about their knowledge of family planning methods, and those who have ever heard about family planning will be asked about their use of family planning methods. Information on sexual and reproductive health information and education will also be collected. Most questions will be close-ended.

The questionnaire will be imported into the REDCap (Research Electronic Data Capture) platform (see https://projectredcap.org/software/)—a software used to design and collect electronic data using electronic gadgets such as mobile phones or tablet computers. This software allows the data to be collected and saved automatically on the device and uploaded unto an online server using internet while on the field. This technology will help minimise the potential for data loss which is common in the context of paper-based questionnaire, which could easily be destroyed by natural events such as rain or fire. Similarly, the automatic saving of the data makes it needless for a separate data entry phase common when paper-based questionnaires are used. However, poor internet access could affect the use of this technology. Therefore, we will procure high speed internet data to support this electronic data collection.

### Quality control measures

In order to ensure data quality, a number of measures will be implemented. All research assistants will be trained by the research team prior to data collection. During the training session, the research assistants will be taken through several aspects of the research and data collection tool, including explaining the main objectives of the study and the data collection techniques. During the training, the research assistants will be made to translate the questions into the relevant local interview language (i.e. *Dagbaani, Gonja and Moar*) to facilitate better understanding of the questions. Furthermore, the research assistants will undertake role-play exercises to help them have a better understanding of the questionnaires, and how to ask the questions appropriately during the actual data collection.

Following the training, the questionnaire will be pre-tested by the research assistants. The pre-test will help reveal likely problems that could arise during the actual data collection. It will also enable the research team to reframe unclear questions and to finalize the questionnaire before the start of actual data collection. In total, the questionnaire will be administered to 20 respondents (5 each in study district) in the pretest.

In addition, the research team will be actively involved in supervising the research assistants at each stage of the data collection to ensure that the data collection is properly done. Furthermore, the REDCap software that will be used for the data collection has inbuilt checks and branching logics that help to reduce data collection, entry and transmission errors.

### Data processing and analysis

Data from the online REDCap platform will be downloaded and exported as excel files. All data files will then be exported into STATA version 15 for cleaning, coding and possible recoding and analysis. Data cleaning will be done by identifying outliers/anomalies and checking for consistency among and across variables. Frequency distributions and cross tabulations will specifically be run to aid the data cleaning process. Basic descriptive statistical analyses will be performed to describe socio-demographic characteristics of respondents, as well as awareness about, and use of modern family planning/contraceptive methods.

In addition to using descriptive statistics to describe important characteristics and changes in SRH outcomes in each experimental arm, the main statistical analytical method will be Difference-in-Difference Analysis. This analytical technique will allow us to detect the true effect of each intervention in comparison to the control group, while controlling for confounders. The statistical analysis will particularly involve comparison of changes in key outcome variables across all arms of the experiment. In addition, Chi-square tests of association will be performed to understand factors associated with sexual and reproductive health services utilization, including examining association between a number of independent variables (e.g. age, sex, education, and disability type) and dependent variables (e.g. awareness about modern family planning/contraceptives, and use of modern contraceptives). Multiple logistic regression models will then be fitted to examine the relationship between dependent variables (e.g. modern contraceptive use) and independent variables. Furthermore, for continuous variables such as number of antenatal visits, multiple linear regression models will be fitted. Finally, cost-effectiveness analyses will be done on the three-intervention arms vis a vis changes in key outcome measures to identify which of the three interventions is likely to yield greater impact with lower costs. For all the inferential quantitative analysis, confidence level will be set at 95% and p-value of less than 0.05 will be taken as demonstrating statistical significance.

## Discussion

In recognition of the relative marginalization of many persons with disability across the world, the United Nations Convention on the Rights of Persons with Disabilities guarantees persons with disabilities the fundamental human rights to access “the same range, quality and standard of free or affordable healthcare and programs as provided to other persons, including those in the area of sexual and reproductive health and population-based public health programs” [28]. While this recognition may have contributed to heightening global awareness about the marginalization and rights of persons with disabilities, available evidence suggests that persons with disabilities still face numerous challenges in accessing and utilizing essential reproductive health services [3] and this affects their quality of life [3, 5–15]. Impediments to receiving required health services include attitudinal biases of health and social service providers, physical barriers in clinical settings, and poor dissemination of information [5–7]. Lack of access to sexual and reproductive health information and services for women with disability constitutes a form of social exclusion and certainly a violation of their right to appropriate and quality sexual and reproductive health care as provided for in the United Nations Convention on the Rights of Persons with Disabilities [28]. Such exclusion could potentially undermine efforts to achieve equitable or universal healthcare access as envisaged in Sustainable Development Goal 3. It is within this context that the study herein proposed becomes relevant. Through this study, we aim to generate research evidence to support effort to achieve universal access to health. This research is also expected to help identify determinants of sexual and reproductive health services utilization as well as barriers to accessibility of sexual and reproductive health information and services currently being provided by both the public and private health sectors in Ghana. It will generate important evidence to inform local health policies/programmes to increase access to sexual and reproductive health among women with disability and as well strengthen local health system capacity to provide disability-sensitive sexual and reproductive healthcare services. In particular, if the proposed interventions are shown to be effective, same or similar interventions could be deployed in similar settings in Ghana and Africa to bridge the reproductive health inequality gap between women with disabilities and women without disability.

The proposed study however has potential limitations. The first is related to recruitment and retention of participants over the 12 months study period. We anticipate challenges in identifying women with disabilities due to stigma associated with disability. To address this challenge, we have proposed the use of community health volunteers who are usual community members to help bridge the gap in identifying women/girls with disabilities in the community. The second limitation is related to the quasi-experimental design of the study. Generally, the evidence from a cluster randomized clinical trial for example is at a much higher level than a quasi-experimental study. However, participants in this study will not be randomly assigned to the interventions as in a randomized control trial. Similarly, participants in the control group will not receive any of the interventions. We note however that participants in the four districts are socially, culturally and economically similar and this similarity will ensure comparability of results. Finally, 12 months may not be sufficiently long enough to expect dramatic positive changes in sexual and reproductive health outcomes of women with disabilities in a context like northern Ghana, where reproductive health outcomes in the general population are already poor.

## Data Availability

All relevant data are included in this paper.

## Abbreviations

ANC: Antenatal care
CHAG: Christian Health Association of Ghana
CHVs: Community health volunteers
DHS: Demographic and Health Survey
FP: Family planning
GFD: Ghana Federation of Disability Organisations
GHS: Ghana Health Service
MoH: Ministry of Health
PWDs: Persons with disabilities
SDGs: Sustainable Development Goals
SRH: Sexual and Reproductive Health
SRHRs: Sexual and reproductive health and rights
SSA: Sub-Saharan Africa
TBAs: Traditional birth attendants
UNCRPWD: United Nations’ Convention on the Rights of Persons with Disabilities
WWDs: Women with disabilities
